# (*E*)-1-[1-(3-Chloro­phen­yl)ethyl­idene]-2-(2,4-dinitro­phen­yl)hydrazine

**DOI:** 10.1107/S160053681200548X

**Published:** 2012-02-17

**Authors:** Hoong-Kun Fun, Suchada Chantrapromma, Boonlerd Nilwanna, Chatchanok Karalai

**Affiliations:** a X-ray Crystallography Unit, School of Physics, Universiti Sains Malaysia, 11800 USM, Penang, Malaysia; b Crystal Materials Research Unit, Department of Chemistry, Faculty of Science, Prince of Songkla University, Hat-Yai, Songkhla 90112, Thailand

## Abstract

There are two crystallographically independent mol­ecules in the asymmetric unit of the title compound, C_14_H_11_ClN_4_O_4_, with the same *E* conformation about the C=N double bond. The mol­ecules are approximately planar, with a dihedral angle between the benzene rings of 10.24 (12)° in one mol­ecule and 4.73 (12)° in the other. In both mol­ecules, the *ortho*-nitro groups of the 2,4-dinitro­phenyl units are coplanar to their bound benzene rings, whereas the *para*-nitro groups are slightly twisted. In each mol­ecule, intra­molecular N—H⋯O hydrogen bonds generate *S*(6) ring motifs. In the crystal, mol­ecules are linked by weak C—H⋯O inter­actions into sheets parallel to the (-102) plane. These sheets are stacked by π–π inter­actions, with centroid–centroid distances of 3.7008 (14) and 3.7459 (14) Å. A Cl⋯O short contact [3.111 (2) Å] is observed.

## Related literature

For bond-length data, see: Allen *et al.* (1987 ▶). For related literature on hydrogen-bond motifs, see: Bernstein *et al.* (1995 ▶). For related structures, see: Chantrapromma *et al.* (2011 ▶); Fun *et al.* (2011 ▶, 2012 ▶); Nilwanna *et al.* (2011 ▶). For background to biological activities of hydrazones, see: Angelusiu *et al.* (2010 ▶); Cui *et al.* (2010 ▶); Gokce *et al.* (2009 ▶); Khan *et al.* (2007 ▶): Loncle *et al.* (2004 ▶); Wang *et al.* (2009 ▶).
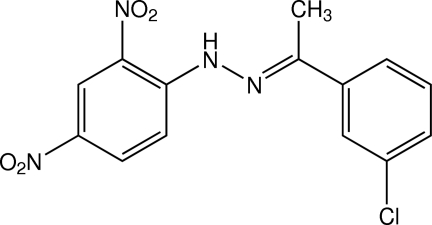


## Experimental

### 

#### Crystal data

C_14_H_11_ClN_4_O_4_
*M* *_r_* = 334.72
Monoclinic, 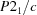

*a* = 13.4825 (13) Å
*b* = 15.1586 (15) Å
*c* = 16.1281 (12) Å
β = 116.815 (6)°
*V* = 2941.8 (5) Å^3^
*Z* = 8
Mo *K*α radiation
μ = 0.29 mm^−1^
*T* = 296 K
0.42 × 0.19 × 0.18 mm

#### Data collection

Bruker APEX DUO CCD area-detector diffractometer
Absorption correction: multi-scan (*SADABS*; Bruker, 2009 ▶) *T*_min_ = 0.889, *T*_max_ = 0.951
32600 measured reflections
8629 independent reflections
4617 reflections with *I* > 2σ(*I*)
*R*_int_ = 0.034

#### Refinement

*R*[*F* ^2^ > 2σ(*F* ^2^)] = 0.051
*wR*(*F* ^2^) = 0.183
*S* = 1.01
8629 reflections
417 parameters
1 restraint
H-atom parameters constrained
Δρ_max_ = 0.35 e Å^−3^
Δρ_min_ = −0.34 e Å^−3^

### 

Data collection: *APEX2* (Bruker, 2009 ▶); cell refinement: *SAINT* (Bruker, 2009 ▶); data reduction: *SAINT*; program(s) used to solve structure: *SHELXTL* (Sheldrick, 2008 ▶); program(s) used to refine structure: *SHELXTL*; molecular graphics: *SHELXTL*; software used to prepare material for publication: *SHELXTL* and *PLATON* (Spek, 2009 ▶).

## Supplementary Material

Crystal structure: contains datablock(s) global, I. DOI: 10.1107/S160053681200548X/rz2707sup1.cif

Structure factors: contains datablock(s) I. DOI: 10.1107/S160053681200548X/rz2707Isup2.hkl

Supplementary material file. DOI: 10.1107/S160053681200548X/rz2707Isup3.cml

Additional supplementary materials:  crystallographic information; 3D view; checkCIF report

## Figures and Tables

**Table 1:** Hydrogen-bond geometry (Å, °)

*D*—H⋯*A*	*D*—H	H⋯*A*	*D*⋯*A*	*D*—H⋯*A*
N1*A*—H1N*A*⋯O1*A*	0.82	1.95	2.598 (2)	135
N1*B*—H1N*B*⋯O1*B*	0.86	1.86	2.589 (2)	141
C5*A*—H5*A*⋯O1*A*^i^	0.93	2.52	3.251 (3)	136
C5*B*—H5*B*⋯O1*B*^ii^	0.93	2.33	3.196 (3)	154
C11*A*—H11*A*⋯O3*B*^iii^	0.93	2.55	3.402 (3)	153
C11*B*—H11*B*⋯O3*A*^iv^	0.93	2.58	3.429 (3)	153

Symmetry codes: (i) 
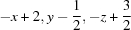
; (ii) 
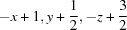
; (iii) 
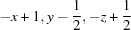
; (iv) 
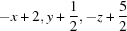
.

**Fig. 1.**
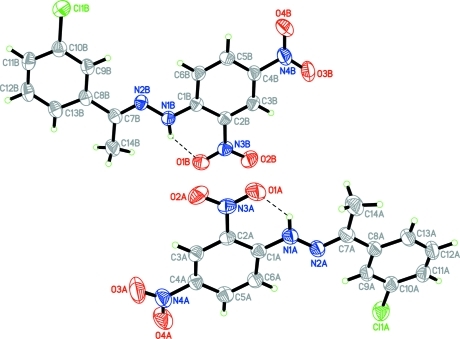
The molecular structure of (I), showing 50% probability displacement ellipsoids. Intramolecular N—H···O hydrogen bonds are shown as dashed lines.

**Fig. 2.**
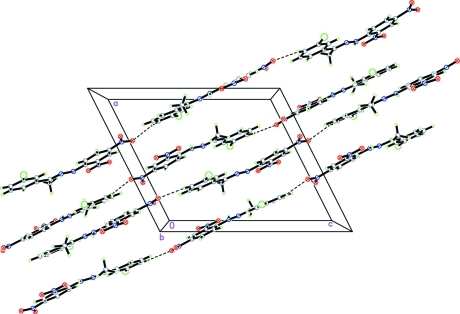
The crystal packing of (I) viewed approximately along the b axis. Hydrogen bonds are shown as dashed lines.

## supplementary crystallographic information

### Comment

Hydrazones are known to be bioactive compounds with antibacterial, antifungal, antitumor, anti-inflammatory as well as antioxidant (Angelusiu *et al.*, 2010; Cui *et al.*, 2010; Gokce *et al.*, 2009; Khan *et al.*, 2007; Loncle *et al.*, 2004; Wang *et al.*, 2009) activities. Within our on-going research on the bioactivity of hydrazones, the title compound (I) was synthesized in order to study and compare its biological activity with other related compounds (Chantrapromma *et al.*, 2011; Fun *et al.*, 2011; 2012; Nilwanna *et al.*, 2011). Herein we report the synthesis and crystal structure of (I).

There are two crystallographic independent molecules *A* and *B* in the asymmetric unit of (I) with differences in bond angles (Fig. 1). The molecular structure of (I) is nearly planar with the dihedral angle between the two benzene rings of 10.24 (12)° in molecule *A* and 4.73 (12)° in molecule *B*. The central ethylidenehydrazine bridge (N1/N2/C7/C14) is planar with the torsion angles N1–N2–C7–C14 = 0.8 (3) and 0.5 (3)° in molecules *A* and *B*, repectively. The mean plane through this central bridge makes dihedral angles of 6.36 (17) and 3.90 (18)° with the 2,4-dinitrophenyl and 3-chlorophenyl rings, respectively in molecule *A* whereas the corresponding values are 5.37 (15) and 0.90 (15)° in molecule *B*. In both molecules, the *ortho*-nitro group of the 2,4-dinitrophenyl is coplanar with the attached benzene ring with the *r.m.s*. deviation of 0.0164 (2) Å for the nine non H-atoms (C1–C6/N3/O1/O2), and torsion angles O1–N3–C2–C3 = 176.84 (18)° and O2–N3–C2–C3 = -1.9 (3)°, whereas the *para*-nitro group is slightly twisted with the torsion angles O3–N4–C4–C5 = 168.8 (2)° and O4–N4–C4–C5 = -11.5 (3)° in molecule *A*; the corresponding values are 0.0176 (2) Å, -177.77 (18), 3.6 (3), 171.1 (2) and -8.9 (4)° in molecule *B*. In each molecule, intramolecular N—H···O hydrogen bond (Fig.1 and Table 1) generates S(6) ring motifs (Bernstein *et al.*, 1995) The bond distances agree with the literature values (Allen *et al.*, 1987) and are comparable with the related structures (Chantrapromma *et al.*, 2011; Fun *et al.*, 2011; 2012; Nilwanna *et al.*, 2011).

In the crystal packing (Fig. 2), the molecules are linked by weak C—H···O interactions (Table 1) into sheets parallel to the (-102) plane. These sheets are further stacked along the *a* axis by π–π interactions with distances of Cg_1_···Cg_2_^v^ = 3.7459 (14) Å and Cg_1_···Cg_3_^vi^ = 3.7008 (14) Å [symmetry codes (v) = x, 3/2-y, 1/2+z; (vi) = 2-x, 2-y, 2-z]; Cg_1_, Cg_2_ and Cg_3_ are the centroids of C1A–C6A, C8A–C13A and C8B–C13B benzene rings, respectively. A Cl1B···O1B^ii^ [3.111 (2) Å] short contact is observed.

### Experimental

The title compound (I) was synthesized by dissolving 2,4-dinitrophenylhydrazine (0.40 g, 2 mmol) in ethanol (10.00 ml) and H_2_SO_4_ (conc.) (98 %, 0.50 ml) was slowly added with stirring. 3-Chloroacetophenone (0.26 ml, 2 mmol) was then added to the solution with continuous stirring. The solution was stirred for 1 h yielding an orange solid, which was filtered off and washed with methanol. Orange block-shaped single crystals of the title compound suitable for X-ray structure determination were recrystalized from ethanol by slow evaporation of the solvent at room temperature over several days. M.p. 478-479 K.

### Refinement

All H atoms were positioned geometrically and allowed to ride on their parent atoms, with d(N-H) = 0.83 and 0.86 Å, d(C-H) = 0.93 Å for aromatic and 0.96 Å for CH_3_ atoms. The *U*_iso_ values were constrained to be 1.5*U*_eq_ of the carrier atom for methyl H atoms and 1.2*U*_eq_ for the remaining H atoms. A rotating group model was used for the methyl groups. A DFIX restraint of 2.00 (1) Å was used for the H14D···H1NB distance. An outlier (0 2 0) was omitted.

### Crystal data

**Table:**

C_14_H_11_ClN_4_O_4_	*F*(000) = 1376
*M**_r_* = 334.72	*D*_x_ = 1.512 Mg m^−^^3^
Monoclinic, *P*2_1_/*c*	Melting point = 478–479 K
Hall symbol: -P 2ybc	Mo *K*α radiation, λ = 0.71073 Å
*a* = 13.4825 (13) Å	Cell parameters from 8629 reflections
*b* = 15.1586 (15) Å	θ = 1.7–30.1°
*c* = 16.1281 (12) Å	µ = 0.29 mm^−^^1^
β = 116.815 (6)°	*T* = 296 K
*V* = 2941.8 (5) Å^3^	Block, orange
*Z* = 8	0.42 × 0.19 × 0.18 mm

### Data collection

**Table:**

Bruker APEX DUO CCD area-detector diffractometer	8629 independent reflections
Radiation source: sealed tube	4617 reflections with *I* > 2σ(*I*)
Graphite monochromator	*R*_int_ = 0.034
φ and ω scans	θ_max_ = 30.1°, θ_min_ = 1.7°
Absorption correction: multi-scan (*SADABS*; Bruker, 2009)	*h* = −18→18
*T*_min_ = 0.889, *T*_max_ = 0.951	*k* = −21→19
32600 measured reflections	*l* = −22→22

### Refinement

**Table:**

Refinement on *F*^2^	Primary atom site location: structure-invariant direct methods
Least-squares matrix: full	Secondary atom site location: difference Fourier map
*R*[*F*^2^ > 2σ(*F*^2^)] = 0.051	Hydrogen site location: inferred from neighbouring sites
*wR*(*F*^2^) = 0.183	H-atom parameters constrained
*S* = 1.01	*w* = 1/[σ^2^(*F*_o_^2^) + (0.0916*P*)^2^ + 0.289*P*] where *P* = (*F*_o_^2^ + 2*F*_c_^2^)/3
8629 reflections	(Δ/σ)_max_ = 0.001
417 parameters	Δρ_max_ = 0.35 e Å^−^^3^
1 restraint	Δρ_min_ = −0.34 e Å^−^^3^

### Special details

**Table:**

Geometry. All esds (except the esd in the dihedral angle between two l.s. planes) are estimated using the full covariance matrix. The cell esds are taken into account individually in the estimation of esds in distances, angles and torsion angles; correlations between esds in cell parameters are only used when they are defined by crystal symmetry. An approximate (isotropic) treatment of cell esds is used for estimating esds involving l.s. planes.
Refinement. Refinement of F^2^ against ALL reflections. The weighted R-factor wR and goodness of fit S are based on F^2^, conventional R-factors R are based on F, with F set to zero for negative F^2^. The threshold expression of F^2^ > 2sigma(F^2^) is used only for calculating R-factors(gt) etc. and is not relevant to the choice of reflections for refinement. R-factors based on F^2^ are statistically about twice as large as those based on F, and R- factors based on ALL data will be even larger.

### Fractional atomic coordinates and isotropic or equivalent isotropic displacement parameters (Å^2^)

**Table:**

	*x*	*y*	*z*	*U*_iso_*/*U*_eq_	
Cl1A	0.83654 (6)	0.45629 (4)	0.39055 (4)	0.0766 (2)	
O1A	0.99997 (15)	0.97920 (10)	0.69823 (12)	0.0774 (5)	
O2A	1.06555 (14)	0.99861 (10)	0.84524 (12)	0.0711 (5)	
O3A	1.2204 (2)	0.76410 (15)	1.05861 (12)	0.1103 (8)	
O4A	1.18161 (18)	0.63048 (13)	1.01190 (12)	0.0949 (6)	
N1A	0.97877 (14)	0.82236 (11)	0.62719 (11)	0.0553 (4)	
H1NA	0.9589	0.8744	0.6200	0.066*	
N2A	0.94483 (14)	0.76046 (11)	0.55856 (11)	0.0536 (4)	
N3A	1.03978 (14)	0.95052 (11)	0.77797 (13)	0.0545 (4)	
N4A	1.18006 (17)	0.70917 (15)	0.99701 (13)	0.0688 (5)	
C1A	1.02714 (15)	0.79618 (12)	0.71746 (13)	0.0457 (4)	
C2A	1.05891 (15)	0.85647 (12)	0.79264 (13)	0.0449 (4)	
C3A	1.10886 (15)	0.82829 (13)	0.88368 (13)	0.0481 (4)	
H3A	1.1298	0.8685	0.9322	0.058*	
C4A	1.12712 (16)	0.73977 (13)	0.90130 (13)	0.0502 (5)	
C5A	1.09807 (17)	0.67883 (13)	0.82982 (14)	0.0556 (5)	
H5A	1.1124	0.6192	0.8435	0.067*	
C6A	1.04898 (18)	0.70614 (12)	0.74032 (14)	0.0533 (5)	
H6A	1.0293	0.6647	0.6930	0.064*	
C7A	0.90349 (17)	0.78840 (14)	0.47443 (14)	0.0534 (5)	
C8A	0.86585 (16)	0.71840 (15)	0.40185 (13)	0.0514 (5)	
C9A	0.87083 (16)	0.63003 (14)	0.42698 (13)	0.0525 (5)	
H9A	0.8993	0.6143	0.4893	0.063*	
C10A	0.83347 (17)	0.56618 (15)	0.35924 (14)	0.0558 (5)	
C11A	0.79092 (17)	0.58681 (18)	0.26572 (14)	0.0636 (6)	
H11A	0.7651	0.5428	0.2208	0.076*	
C12A	0.78777 (18)	0.67358 (19)	0.24109 (15)	0.0678 (6)	
H12A	0.7610	0.6885	0.1787	0.081*	
C13A	0.82399 (18)	0.73955 (17)	0.30792 (14)	0.0614 (6)	
H13A	0.8203	0.7982	0.2900	0.074*	
C14A	0.8916 (3)	0.88420 (17)	0.44724 (17)	0.0849 (8)	
H14A	0.9563	0.9159	0.4895	0.127*	
H14B	0.8270	0.9082	0.4495	0.127*	
H14C	0.8839	0.8896	0.3853	0.127*	
Cl1B	0.60994 (6)	1.29099 (4)	1.10126 (4)	0.0783 (2)	
O1B	0.58475 (15)	0.77573 (10)	0.80697 (11)	0.0683 (4)	
O2B	0.51645 (18)	0.75501 (10)	0.66003 (13)	0.0860 (6)	
O3B	0.35381 (19)	0.98474 (14)	0.44260 (11)	0.1013 (7)	
O4B	0.3250 (2)	1.11127 (14)	0.48825 (13)	0.1090 (8)	
N1B	0.58812 (13)	0.93100 (11)	0.87512 (11)	0.0490 (4)	
H1NB	0.6001	0.8750	0.8796	0.059*	
N2B	0.60588 (13)	0.99163 (11)	0.94310 (10)	0.0474 (4)	
N3B	0.53830 (15)	0.80306 (11)	0.72640 (13)	0.0556 (4)	
N4B	0.36352 (19)	1.03694 (14)	0.50342 (13)	0.0729 (6)	
C1B	0.53726 (15)	0.95592 (12)	0.78481 (12)	0.0432 (4)	
C2B	0.51113 (15)	0.89601 (11)	0.71062 (13)	0.0445 (4)	
C3B	0.45551 (17)	0.92247 (13)	0.61880 (13)	0.0507 (5)	
H3B	0.4394	0.8822	0.5709	0.061*	
C4B	0.42477 (17)	1.00861 (13)	0.59995 (13)	0.0517 (5)	
C5B	0.45036 (18)	1.07026 (13)	0.67045 (14)	0.0564 (5)	
H5B	0.4293	1.1289	0.6559	0.068*	
C6B	0.50633 (18)	1.04464 (12)	0.76078 (14)	0.0513 (5)	
H6B	0.5245	1.0865	0.8076	0.062*	
C7B	0.64883 (16)	0.96382 (13)	1.02719 (13)	0.0479 (4)	
C8B	0.66519 (15)	1.03272 (14)	1.09812 (13)	0.0475 (4)	
C9B	0.63316 (16)	1.11936 (14)	1.07065 (13)	0.0505 (5)	
H9B	0.6015	1.1343	1.0080	0.061*	
C10B	0.64837 (16)	1.18318 (15)	1.13627 (14)	0.0539 (5)	
C11B	0.69611 (17)	1.16370 (17)	1.22983 (14)	0.0589 (5)	
H11B	0.7068	1.2075	1.2734	0.071*	
C12B	0.72749 (19)	1.07829 (18)	1.25720 (14)	0.0631 (6)	
H12B	0.7595	1.0640	1.3200	0.076*	
C13B	0.71201 (17)	1.01338 (15)	1.19250 (14)	0.0572 (5)	
H13B	0.7332	0.9557	1.2123	0.069*	
C14B	0.6820 (2)	0.86989 (15)	1.05689 (15)	0.0722 (7)	
H14D	0.6413	0.8311	1.0056	0.108*	
H14E	0.6656	0.8557	1.1074	0.108*	
H14F	0.7602	0.8629	1.0766	0.108*	

### Atomic displacement parameters (Å^2^)

**Table:**

	*U*^11^	*U*^22^	*U*^33^	*U*^12^	*U*^13^	*U*^23^
Cl1A	0.0978 (5)	0.0648 (4)	0.0690 (4)	−0.0055 (3)	0.0391 (3)	−0.0079 (3)
O1A	0.1049 (13)	0.0437 (8)	0.0721 (11)	0.0150 (8)	0.0298 (10)	0.0122 (8)
O2A	0.0885 (12)	0.0463 (8)	0.0820 (11)	0.0005 (8)	0.0417 (10)	−0.0156 (8)
O3A	0.159 (2)	0.0944 (15)	0.0468 (10)	−0.0004 (14)	0.0199 (11)	0.0000 (10)
O4A	0.1274 (16)	0.0722 (12)	0.0691 (11)	0.0088 (11)	0.0301 (11)	0.0272 (9)
N1A	0.0674 (11)	0.0427 (9)	0.0489 (9)	0.0047 (8)	0.0200 (8)	0.0013 (7)
N2A	0.0591 (10)	0.0508 (9)	0.0439 (9)	0.0023 (8)	0.0170 (8)	0.0003 (7)
N3A	0.0582 (10)	0.0393 (8)	0.0657 (11)	0.0026 (7)	0.0278 (9)	−0.0008 (8)
N4A	0.0792 (13)	0.0687 (13)	0.0522 (11)	0.0052 (10)	0.0240 (10)	0.0111 (10)
C1A	0.0472 (10)	0.0407 (9)	0.0468 (10)	0.0006 (8)	0.0192 (8)	0.0013 (8)
C2A	0.0481 (10)	0.0346 (9)	0.0529 (10)	0.0007 (7)	0.0236 (8)	−0.0007 (8)
C3A	0.0483 (10)	0.0478 (10)	0.0485 (10)	−0.0041 (8)	0.0223 (8)	−0.0054 (8)
C4A	0.0537 (11)	0.0496 (11)	0.0446 (10)	−0.0004 (9)	0.0199 (9)	0.0039 (8)
C5A	0.0666 (13)	0.0363 (9)	0.0582 (12)	0.0001 (9)	0.0233 (10)	0.0042 (8)
C6A	0.0657 (12)	0.0369 (9)	0.0504 (11)	−0.0002 (8)	0.0199 (9)	−0.0035 (8)
C7A	0.0497 (11)	0.0563 (12)	0.0512 (11)	0.0054 (9)	0.0200 (9)	0.0077 (9)
C8A	0.0441 (10)	0.0649 (13)	0.0442 (10)	0.0063 (9)	0.0192 (8)	0.0061 (9)
C9A	0.0506 (11)	0.0646 (13)	0.0408 (10)	0.0008 (9)	0.0193 (8)	0.0008 (9)
C10A	0.0503 (11)	0.0668 (13)	0.0505 (11)	0.0014 (10)	0.0231 (9)	−0.0014 (10)
C11A	0.0521 (12)	0.0900 (18)	0.0467 (11)	−0.0003 (11)	0.0205 (10)	−0.0109 (11)
C12A	0.0616 (13)	0.0988 (19)	0.0394 (11)	0.0085 (13)	0.0194 (10)	0.0060 (11)
C13A	0.0604 (12)	0.0751 (15)	0.0459 (11)	0.0089 (11)	0.0214 (9)	0.0124 (10)
C14A	0.122 (2)	0.0617 (15)	0.0613 (15)	0.0093 (15)	0.0331 (15)	0.0130 (12)
Cl1B	0.1019 (5)	0.0664 (4)	0.0699 (4)	0.0174 (3)	0.0415 (4)	−0.0006 (3)
O1B	0.0926 (12)	0.0426 (8)	0.0695 (10)	0.0050 (7)	0.0364 (9)	0.0099 (7)
O2B	0.1302 (16)	0.0440 (8)	0.0760 (11)	0.0024 (9)	0.0396 (11)	−0.0144 (8)
O3B	0.159 (2)	0.0877 (14)	0.0431 (9)	0.0147 (13)	0.0329 (11)	0.0020 (9)
O4B	0.162 (2)	0.0778 (13)	0.0645 (11)	0.0374 (14)	0.0311 (12)	0.0214 (10)
N1B	0.0603 (10)	0.0419 (8)	0.0455 (8)	−0.0016 (7)	0.0246 (7)	−0.0001 (7)
N2B	0.0531 (9)	0.0474 (9)	0.0445 (8)	−0.0053 (7)	0.0245 (7)	−0.0007 (7)
N3B	0.0685 (11)	0.0395 (9)	0.0606 (11)	−0.0029 (8)	0.0307 (9)	−0.0032 (8)
N4B	0.0972 (15)	0.0665 (13)	0.0486 (11)	0.0051 (11)	0.0273 (10)	0.0099 (10)
C1B	0.0486 (10)	0.0414 (9)	0.0452 (10)	−0.0051 (7)	0.0260 (8)	0.0002 (7)
C2B	0.0517 (10)	0.0348 (9)	0.0504 (10)	−0.0048 (7)	0.0261 (9)	−0.0017 (7)
C3B	0.0636 (12)	0.0452 (10)	0.0470 (10)	−0.0059 (9)	0.0284 (9)	−0.0049 (8)
C4B	0.0663 (13)	0.0476 (11)	0.0425 (10)	−0.0020 (9)	0.0257 (9)	0.0028 (8)
C5B	0.0775 (14)	0.0387 (10)	0.0562 (12)	0.0016 (9)	0.0331 (11)	0.0065 (9)
C6B	0.0708 (13)	0.0386 (9)	0.0481 (10)	−0.0031 (9)	0.0301 (10)	−0.0033 (8)
C7B	0.0459 (10)	0.0526 (11)	0.0448 (10)	−0.0042 (8)	0.0202 (8)	0.0046 (8)
C8B	0.0440 (10)	0.0578 (12)	0.0419 (10)	−0.0066 (8)	0.0204 (8)	0.0019 (8)
C9B	0.0500 (11)	0.0621 (12)	0.0392 (9)	−0.0009 (9)	0.0200 (8)	0.0025 (9)
C10B	0.0508 (11)	0.0626 (13)	0.0513 (11)	−0.0009 (9)	0.0258 (9)	−0.0022 (9)
C11B	0.0598 (12)	0.0763 (15)	0.0458 (11)	−0.0061 (11)	0.0284 (10)	−0.0090 (10)
C12B	0.0655 (13)	0.0870 (17)	0.0378 (10)	−0.0071 (12)	0.0243 (9)	0.0029 (11)
C13B	0.0603 (12)	0.0664 (13)	0.0451 (10)	−0.0026 (10)	0.0240 (9)	0.0083 (10)
C14B	0.0949 (18)	0.0579 (14)	0.0520 (12)	0.0059 (12)	0.0228 (12)	0.0077 (10)

### Geometric parameters (Å, º)

**Table:**

Cl1A—C10A	1.736 (2)	Cl1B—C10B	1.730 (2)
O1A—N3A	1.228 (2)	O1B—N3B	1.232 (2)
O2A—N3A	1.220 (2)	O2B—N3B	1.216 (2)
O3A—N4A	1.220 (3)	O3B—N4B	1.221 (3)
O4A—N4A	1.215 (3)	O4B—N4B	1.218 (3)
N1A—C1A	1.359 (2)	N1B—C1B	1.354 (2)
N1A—N2A	1.363 (2)	N1B—N2B	1.366 (2)
N1A—H1NA	0.8247	N1B—H1NB	0.8610
N2A—C7A	1.284 (2)	N2B—C7B	1.282 (2)
N3A—C2A	1.449 (2)	N3B—C2B	1.449 (2)
N4A—C4A	1.454 (3)	N4B—C4B	1.460 (3)
C1A—C6A	1.410 (3)	C1B—C6B	1.410 (2)
C1A—C2A	1.421 (3)	C1B—C2B	1.414 (2)
C2A—C3A	1.378 (3)	C2B—C3B	1.384 (3)
C3A—C4A	1.371 (3)	C3B—C4B	1.362 (3)
C3A—H3A	0.9300	C3B—H3B	0.9300
C4A—C5A	1.389 (3)	C4B—C5B	1.390 (3)
C5A—C6A	1.353 (3)	C5B—C6B	1.361 (3)
C5A—H5A	0.9300	C5B—H5B	0.9300
C6A—H6A	0.9300	C6B—H6B	0.9300
C7A—C8A	1.489 (3)	C7B—C8B	1.490 (3)
C7A—C14A	1.505 (3)	C7B—C14B	1.504 (3)
C8A—C9A	1.392 (3)	C8B—C13B	1.391 (3)
C8A—C13A	1.395 (3)	C8B—C9B	1.391 (3)
C9A—C10A	1.374 (3)	C9B—C10B	1.379 (3)
C9A—H9A	0.9300	C9B—H9B	0.9300
C10A—C11A	1.386 (3)	C10B—C11B	1.379 (3)
C11A—C12A	1.369 (4)	C11B—C12B	1.372 (4)
C11A—H11A	0.9300	C11B—H11B	0.9300
C12A—C13A	1.388 (3)	C12B—C13B	1.380 (3)
C12A—H12A	0.9300	C12B—H12B	0.9300
C13A—H13A	0.9300	C13B—H13B	0.9300
C14A—H14A	0.9600	C14B—H14D	0.9600
C14A—H14B	0.9600	C14B—H14E	0.9600
C14A—H14C	0.9600	C14B—H14F	0.9600
			
C1A—N1A—N2A	119.48 (16)	C1B—N1B—N2B	119.72 (16)
C1A—N1A—H1NA	113.5	C1B—N1B—H1NB	110.5
N2A—N1A—H1NA	125.2	N2B—N1B—H1NB	129.6
C7A—N2A—N1A	117.24 (18)	C7B—N2B—N1B	117.36 (17)
O2A—N3A—O1A	122.28 (17)	O2B—N3B—O1B	122.21 (18)
O2A—N3A—C2A	118.97 (18)	O2B—N3B—C2B	119.09 (18)
O1A—N3A—C2A	118.74 (17)	O1B—N3B—C2B	118.69 (16)
O4A—N4A—O3A	123.2 (2)	O4B—N4B—O3B	123.8 (2)
O4A—N4A—C4A	118.6 (2)	O4B—N4B—C4B	118.1 (2)
O3A—N4A—C4A	118.2 (2)	O3B—N4B—C4B	118.1 (2)
N1A—C1A—C6A	120.38 (17)	N1B—C1B—C6B	120.37 (17)
N1A—C1A—C2A	122.72 (17)	N1B—C1B—C2B	122.88 (16)
C6A—C1A—C2A	116.90 (17)	C6B—C1B—C2B	116.75 (17)
C3A—C2A—C1A	121.59 (17)	C3B—C2B—C1B	121.82 (17)
C3A—C2A—N3A	116.37 (17)	C3B—C2B—N3B	116.22 (17)
C1A—C2A—N3A	122.03 (17)	C1B—C2B—N3B	121.94 (17)
C4A—C3A—C2A	118.68 (18)	C4B—C3B—C2B	118.70 (18)
C4A—C3A—H3A	120.7	C4B—C3B—H3B	120.6
C2A—C3A—H3A	120.7	C2B—C3B—H3B	120.6
C3A—C4A—C5A	121.47 (18)	C3B—C4B—C5B	121.59 (18)
C3A—C4A—N4A	119.25 (19)	C3B—C4B—N4B	119.28 (18)
C5A—C4A—N4A	119.27 (19)	C5B—C4B—N4B	119.14 (19)
C6A—C5A—C4A	120.08 (18)	C6B—C5B—C4B	119.81 (19)
C6A—C5A—H5A	120.0	C6B—C5B—H5B	120.1
C4A—C5A—H5A	120.0	C4B—C5B—H5B	120.1
C5A—C6A—C1A	121.27 (18)	C5B—C6B—C1B	121.28 (18)
C5A—C6A—H6A	119.4	C5B—C6B—H6B	119.4
C1A—C6A—H6A	119.4	C1B—C6B—H6B	119.4
N2A—C7A—C8A	115.29 (19)	N2B—C7B—C8B	114.92 (18)
N2A—C7A—C14A	124.4 (2)	N2B—C7B—C14B	125.14 (19)
C8A—C7A—C14A	120.33 (19)	C8B—C7B—C14B	119.94 (17)
C9A—C8A—C13A	118.7 (2)	C13B—C8B—C9B	118.04 (19)
C9A—C8A—C7A	120.14 (17)	C13B—C8B—C7B	121.96 (19)
C13A—C8A—C7A	121.2 (2)	C9B—C8B—C7B	120.00 (17)
C10A—C9A—C8A	119.60 (19)	C10B—C9B—C8B	120.03 (18)
C10A—C9A—H9A	120.2	C10B—C9B—H9B	120.0
C8A—C9A—H9A	120.2	C8B—C9B—H9B	120.0
C9A—C10A—C11A	122.0 (2)	C11B—C10B—C9B	121.6 (2)
C9A—C10A—Cl1A	119.52 (16)	C11B—C10B—Cl1B	118.84 (18)
C11A—C10A—Cl1A	118.49 (18)	C9B—C10B—Cl1B	119.55 (16)
C12A—C11A—C10A	118.4 (2)	C12B—C11B—C10B	118.6 (2)
C12A—C11A—H11A	120.8	C12B—C11B—H11B	120.7
C10A—C11A—H11A	120.8	C10B—C11B—H11B	120.7
C11A—C12A—C13A	120.9 (2)	C11B—C12B—C13B	120.7 (2)
C11A—C12A—H12A	119.6	C11B—C12B—H12B	119.7
C13A—C12A—H12A	119.6	C13B—C12B—H12B	119.7
C12A—C13A—C8A	120.4 (2)	C12B—C13B—C8B	121.1 (2)
C12A—C13A—H13A	119.8	C12B—C13B—H13B	119.5
C8A—C13A—H13A	119.8	C8B—C13B—H13B	119.5
C7A—C14A—H14A	109.5	C7B—C14B—H14D	109.5
C7A—C14A—H14B	109.5	C7B—C14B—H14E	109.5
H14A—C14A—H14B	109.5	H14D—C14B—H14E	109.5
C7A—C14A—H14C	109.5	C7B—C14B—H14F	109.5
H14A—C14A—H14C	109.5	H14D—C14B—H14F	109.5
H14B—C14A—H14C	109.5	H14E—C14B—H14F	109.5
			
C1A—N1A—N2A—C7A	−177.38 (18)	C1B—N1B—N2B—C7B	176.29 (17)
N2A—N1A—C1A—C6A	4.3 (3)	N2B—N1B—C1B—C6B	2.4 (3)
N2A—N1A—C1A—C2A	−176.33 (18)	N2B—N1B—C1B—C2B	−176.75 (17)
N1A—C1A—C2A—C3A	−179.36 (18)	N1B—C1B—C2B—C3B	177.62 (18)
C6A—C1A—C2A—C3A	0.1 (3)	C6B—C1B—C2B—C3B	−1.5 (3)
N1A—C1A—C2A—N3A	1.2 (3)	N1B—C1B—C2B—N3B	−0.6 (3)
C6A—C1A—C2A—N3A	−179.35 (18)	C6B—C1B—C2B—N3B	−179.79 (17)
O2A—N3A—C2A—C3A	−1.9 (3)	O2B—N3B—C2B—C3B	3.6 (3)
O1A—N3A—C2A—C3A	176.84 (18)	O1B—N3B—C2B—C3B	−177.77 (18)
O2A—N3A—C2A—C1A	177.57 (18)	O2B—N3B—C2B—C1B	−178.10 (19)
O1A—N3A—C2A—C1A	−3.7 (3)	O1B—N3B—C2B—C1B	0.6 (3)
C1A—C2A—C3A—C4A	−0.5 (3)	C1B—C2B—C3B—C4B	−0.4 (3)
N3A—C2A—C3A—C4A	178.92 (17)	N3B—C2B—C3B—C4B	177.93 (18)
C2A—C3A—C4A—C5A	1.0 (3)	C2B—C3B—C4B—C5B	1.6 (3)
C2A—C3A—C4A—N4A	179.80 (18)	C2B—C3B—C4B—N4B	−178.42 (19)
O4A—N4A—C4A—C3A	169.6 (2)	O4B—N4B—C4B—C3B	171.1 (2)
O3A—N4A—C4A—C3A	−10.0 (3)	O3B—N4B—C4B—C3B	−8.9 (3)
O4A—N4A—C4A—C5A	−11.5 (3)	O4B—N4B—C4B—C5B	−8.9 (4)
O3A—N4A—C4A—C5A	168.8 (2)	O3B—N4B—C4B—C5B	171.1 (2)
C3A—C4A—C5A—C6A	−1.1 (3)	C3B—C4B—C5B—C6B	−0.8 (3)
N4A—C4A—C5A—C6A	−179.8 (2)	N4B—C4B—C5B—C6B	179.3 (2)
C4A—C5A—C6A—C1A	0.6 (3)	C4B—C5B—C6B—C1B	−1.3 (3)
N1A—C1A—C6A—C5A	179.35 (19)	N1B—C1B—C6B—C5B	−176.77 (19)
C2A—C1A—C6A—C5A	−0.1 (3)	C2B—C1B—C6B—C5B	2.4 (3)
N1A—N2A—C7A—C8A	−179.01 (17)	N1B—N2B—C7B—C8B	−179.42 (15)
N1A—N2A—C7A—C14A	0.8 (3)	N1B—N2B—C7B—C14B	0.5 (3)
N2A—C7A—C8A—C9A	3.7 (3)	N2B—C7B—C8B—C13B	−179.25 (18)
C14A—C7A—C8A—C9A	−176.1 (2)	C14B—C7B—C8B—C13B	0.8 (3)
N2A—C7A—C8A—C13A	−177.09 (19)	N2B—C7B—C8B—C9B	0.8 (3)
C14A—C7A—C8A—C13A	3.1 (3)	C14B—C7B—C8B—C9B	−179.11 (19)
C13A—C8A—C9A—C10A	−0.8 (3)	C13B—C8B—C9B—C10B	0.1 (3)
C7A—C8A—C9A—C10A	178.49 (18)	C7B—C8B—C9B—C10B	−179.94 (18)
C8A—C9A—C10A—C11A	0.3 (3)	C8B—C9B—C10B—C11B	0.7 (3)
C8A—C9A—C10A—Cl1A	−178.40 (15)	C8B—C9B—C10B—Cl1B	179.10 (15)
C9A—C10A—C11A—C12A	0.8 (3)	C9B—C10B—C11B—C12B	−0.9 (3)
Cl1A—C10A—C11A—C12A	179.51 (17)	Cl1B—C10B—C11B—C12B	−179.32 (17)
C10A—C11A—C12A—C13A	−1.4 (3)	C10B—C11B—C12B—C13B	0.3 (3)
C11A—C12A—C13A—C8A	0.9 (3)	C11B—C12B—C13B—C8B	0.5 (3)
C9A—C8A—C13A—C12A	0.2 (3)	C9B—C8B—C13B—C12B	−0.7 (3)
C7A—C8A—C13A—C12A	−179.1 (2)	C7B—C8B—C13B—C12B	179.35 (19)

### Hydrogen-bond geometry (Å, º)

**Table:**

*D*—H···*A*	*D*—H	H···*A*	*D*···*A*	*D*—H···*A*
N1*A*—H1*NA*···O1*A*	0.82	1.95	2.598 (2)	135
N1*B*—H1*NB*···O1*B*	0.86	1.86	2.589 (2)	141
C5*A*—H5*A*···O1*A*^i^	0.93	2.52	3.251 (3)	136
C5*B*—H5*B*···O1*B*^ii^	0.93	2.33	3.196 (3)	154
C11*A*—H11*A*···O3*B*^iii^	0.93	2.55	3.402 (3)	153
C11*B*—H11*B*···O3*A*^iv^	0.93	2.58	3.429 (3)	153

Symmetry codes: (i) −*x*+2, *y*−1/2, −*z*+3/2; (ii) −*x*+1, *y*+1/2, −*z*+3/2; (iii) −*x*+1, *y*−1/2, −*z*+1/2; (iv) −*x*+2, *y*+1/2, −*z*+5/2.
